# Feasibility and impact of a computerised clinical decision support system on investigation and initial management of new onset chest pain: a mixed methods study

**DOI:** 10.1186/s12911-015-0189-8

**Published:** 2015-08-26

**Authors:** Rachel Johnson, Maggie Evans, Helen Cramer, Kristina Bennert, Richard Morris, Sandra Eldridge, Katy Juttner, Mohammed J Zaman, Harry Hemingway, Spiros Denaxas, Adam Timmis, Gene Feder

**Affiliations:** Centre for Academic Primary Care, School of Social and Community Medicine, University of Bristol, Bristol, UK; School of Social and Community Medicine, University of Bristol, Bristol, UK; Department of Primary Care & Population Health, University College London, London, UK; Centre for Primary Care and Public Health Queen Mary, University of London, London, UK; Bedminster Family Practice, Bristol, UK; James Paget University Hospital, Norfolk, UK; Farr Institute of Health Informatics Research London, Institute of Health Informatics, University College London, London, UK; NIHR Cardiovascular Biomedical Research Unit, Barts Health NHS Trust, London, UK

**Keywords:** Chest pain, Angina, Diagnosis, Computerised decision support system, Qualitative, Mixed methods, Clinical guideline

## Abstract

**Background:**

Clinical decision support systems (CDSS) can modify clinician behaviour, yet the factors influencing their effect remain poorly understood. This study assesses the feasibility and acceptability of a CDSS supporting diagnostic and treatment decisions for patients with suspected stable angina.

**Methods:**

*Intervention* The Optimising Management of Angina (OMA) programme includes a CDSS guiding investigation and medication decisions for clinicians managing patients with new onset stable angina, based on English national guidelines, introduced through an educational intervention. *Design and participants* A mixed methods study *i.* A study of outcomes among patients presenting with suspected angina in three chest pain clinics in England before and after introduction of the OMA programme. ii. Observations of clinic processes, interviews and a focus group with health professionals at two chest pain clinics after delivery of the OMA programme. *Outcomes*. Medication and cardiovascular imaging investigations undertaken within six months of presentation, and concordance of these with the recommendations of the CDSS. Thematic analysis of qualitative data to understand how the CDSS was used.

**Results:**

Data were analysed for 285 patients attending chest pain clinics: 106 before and 179 after delivery of the OMA programme. 40 consultations were observed, 5 clinicians interviewed, and a focus group held after the intervention. The proportion of patients appropriate for diagnostic investigation who received one was 50 % (95 CI 34–66 %) of those before OMA and 59 % (95 CI 48–70 %) of those after OMA. Despite high use of the CDSS (84 % of consultations), observations and interviews revealed difficulty with data entry into the CDSS, and structural and practical barriers to its use. In the majority of cases the CDSS was not used to guide real-time decision making, only being consulted after the patient had left the room.

**Conclusions:**

The OMA CDSS for the management of chest pain is not feasible in its current form. The CDSS was not used to support decisions about the care of individual patients. A range of barriers to the use of the CDSS were identified, some are easily removed, such as insufficient capture of cardiovascular risk, while others are more deeply embedded in current practice, such as unavailability of some investigations or no prescribing privileges for nurses.

## Background

Clinicians need to use the best available evidence to inform patient management decisions. Clinical decision support systems (CDSSs) generate patient-specific assessments or recommendations to aid clinical decision making. CDSSs can improve practitioner performance and health care processes in a range of clinical scenarios including disease management, drug-dosing, prescribing and preventive care [1–3]. The effect of CDSSs on clinical outcomes is less frequently studied. In a systematic review of 148 randomised controlled trials of CDSSs implemented in clinical settings only 29 assessed clinical outcomes [1]. There was moderate evidence in support of an effect of CDSSs on morbidity from 16 studies; evidence for other clinical outcomes was of low quality.

Few studies have sought rigorous evidence to determine what factors contribute to the effectiveness of decision support systems [4]. A recent meta-regression of computerised CDSSs found sufficient quality evidence for the assessment of only six factors. Of these, success of CDSSs was positively associated with systems developed by the investigators of the primary studies, systems providing advice to patients and practitioners, and systems requiring a reason for overriding advice [5]. It has been argued that research based on randomised controlled trials is not sufficient to allow rigorous assessment of the factors that make CDSSs successful, and that qualitative contextual evaluation and observation are needed [4].

In the United Kingdom (UK), initial specialist assessment of suspected angina is undertaken in chest pain clinics. Chest pain clinics may incorrectly diagnose some patients, and may not initiate secondary prevention in those with angina. In a cohort of 8762 patients followed for three years after attending a chest pain clinic, 33 % of all cardiovascular events occurred in patients with a diagnosis of non-cardiac chest pain [6]. This study reported under-prescription of effective secondary prevention: of those diagnosed with angina only 28 % were taking a statin [7].

Our Optimising the Management of Angina (OMA) programme includes a web-based computerised CDSS to support investigation and medication decisions for patients with new onset stable chest pain. We hypothesised that appropriate investigation, leading to accurate diagnosis and effective secondary prevention, would lead to a reduction in cardiovascular events. Here we present a mixed methods study that aimed to understand the factors influencing the feasibility of the OMA CDSS in clinical practice, and the impact of the CDSS on investigation and prescribing behaviour, to inform potential progression to a cluster randomised trial of the intervention.

## Methods

**Fig. 1:**
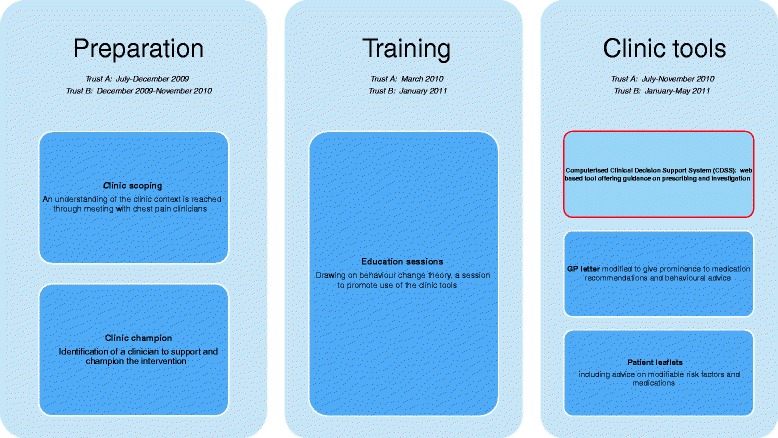
The OMA programme. Preparation and Training stages facilitate the use of three clinic tools (CDSS, GP letter and patient leaflets)

The OMA programme is a complex intervention delivered at the level of the clinic. The programme has 3 stages: *preparation, training* and *clinic tools* (Fig. 1). The *preparation* and *training* stages facilitate use of the OMA CDSS. The components of the intervention were developed using behavioural change domains identified by Michie and colleagues [8].

## Design

We used mixed methods, combining a before-and-after study of chest pain clinic investigation and treatment decisions with observations of clinical practice, interviews and a focus group. In this paper we report on data collected directly following incorporation of the CDSS into the clinics.

### Setting and study population

Two hospital trusts (Trust A and Trust B) in a provincial city recruited chest pain clinic patients both before and after delivery of the OMA programme. Clinicians with responsibility for the chest pain clinics in each trust were identified and approached by members of the research team. With their agreement a meeting was arranged with cardiology clinicians and managers to explain the planned study and obtain agreement to participate. A clinic champion (senior clinician who agreed to support the intervention) was identified at each trust, and all clinicians working regularly within the clinic were invited to take part in the study. Patients were eligible for the study if they had been referred to the chest pain clinic by their general practitioner. We excluded patients who had a previous history of cardiovascular disease, and those who did not speak English. Dates of delivery of the OMA programme are given in Fig. 1, and study recruitment is summarised in Fig. 3.

All clinicians working regularly in the chest pain clinics gave written informed consent to participate in the qualitative study. Consecutive patients attending the clinic after incorporation of the CDSS were invited to give written, informed consent to their consultations being observed and audio-recorded, and to their medical records being accessed. All participating patients gave written informed consent.

### The OMA CDSS

The OMA CDSS guides investigation and prescribing decisions for patients with new onset chest pain. The CDSS was developed by the research team during 2009/10, and was funded by an NIHR programme grant (RP-PG-0407-19314). During development of the CDSS, researchers sought to understand clinicians’ work practices and decision making within both trusts (Preparation stage). This process included researchers meeting with the clinic champion, semi-structured interviews with all regular chest pain clinicians, and qualitative observation of clinic practices. Following this, at Trust A the prototype CDSS was presented to clinicians, allowing user testing of the CDSS in hypothetical scenarios. Insights from these processes were used to modify the CDSS in line with working practices, and to inform a training session for the finalised CDSS. Initially planned as a CDSS based on expert panel consensus, the CDSS was modified in March 2010 to incorporate newly published UK guidance from the National Institute for Health and Care Excellence (NICE CG95) [9]. This modified CDSS was again discussed with clinicians and feedback sought in a clinician focus group, before final modifications and subsequent testing within the pilot study presented here. Prior to its use in practice, in a training session at each site (training stage) we described the rationale for the CDSS and CDSS recommendations were developed, and facilitated discussion of how to use the tool in practice using case scenarios. Clinicians were invited by email to attend the training session. Publicity for the OMA CDSS was limited to the stages described in this section.

**Fig. 2:**
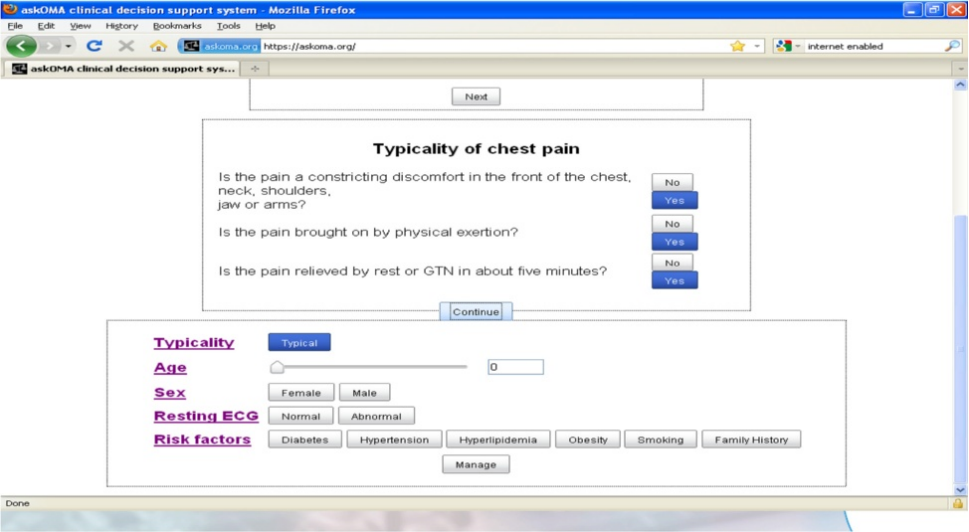
Screenshot of the OMA CDSS clinical data entry page

The CDSS was designed for use by any clinician assessing and managing patients in the chest pain clinic setting. In our study settings clinicians included cardiology specialist nurses, cardiologists and cardiac physiologists. CDSS use was optional for the physicians with no incentives for its use. We were unable to integrate the CDSS with existing electronic systems; clinicians entered data about each patient manually. Clinicians enter clinical information required to calculate a patient’s pre-test probability for coronary heart disease [10], on which investigation and medication recommendations are based (Fig. 2). For individuals with a pre-test probability of coronary disease of <90 % investigation recommendations were based on NICE guidance [9]. The NICE guidelines do not give investigation recommendations for individuals with a pre-test probability of coronary disease of >90 %. A panel of 12 cardiac clinicians agreed that these patients should all have angiography unless there were contraindications to further treatment. CDSS medication recommendations were provided by the panel, and included recommendations for up to three drug classes: anti-platelets, statins and beta-blockers. After receiving recommendations from the CDSS, clinicians were prompted to record their agreement or disagreement with the recommendations and the reasons for these.

### Quantitative data collection and analysis

The following data were extracted from the clinic record at presentation to the clinic: age, sex, cardiovascular risk factors, resting ECG and typicality of chest pain. These data were collected from the CDSS when it had been used, and from the clinic record for all other patients. When the CDSS was not used, two members of the research team (AT and MJZ) labelled the chest pain as typical, atypical or non-cardiac based on the hospital record. Medications (anti-platelets, beta-blockers and statins) current at the clinic date were extracted from the primary care electronic record.

Participants were followed up for six months. Prescriptions of anti-platelet medication, beta-blockers and statins current at six months after the clinic were extracted from the electronic primary care record. Cardiac investigations (computerised tomography, calcium scoring, coronary angiography, stress echocardiography, myocardial perfusion scanning, angiography/angioplasty, cardiac MRI) performed in the six months after the chest pain clinic were collected from the hospital records.

Participants were analysed in two groups: patients seen before delivery of the OMA programme (*Before OMA*) and patients seen after delivery of the OMA programme (*After OMA)*. Concordance with the CDSS recommendation of investigations done within six months and medications prescribed at six months was determined for the *After OMA* group. For patients for whom the CDSS was not available (*Before OMA*) or not used (*After OMA*), we used data from the clinic record to generate CDSS recommendations, in order to determine whether the investigations done and medications prescribed were in line with what the CDSS would have recommended. If we assume that CDSS recommendations would be followed for 75 % of patients even without the use of OMA, and that this percentage would increase to 85 % when OMA was used, we would be required to study 354 patients with and without use of OMA (708 in all) to detect this difference as statistically significant at 5 % level, with 90 % power. As this was a feasibility study, our sample size was based on pragmatic considerations: the number of patients consulting at the chest pain clinic over the course of the data collection phase of the study. We report only descriptive statistics, with 95 % confidence intervals to convey the precision of estimates.

### Qualitative data collection and analysis

We conducted a qualitative study in Trust A during the four months following delivery of the OMA programme. This included chest pain clinic observations, followed by face-to-face interviews with clinicians, and a focus group with clinicians after the CDSS had been in use for three months. Observation enabled systematic inquiry into the nature and quality of observable behaviours, in particular how the CDSS was incorporated into clinic practice. Brief interviews with clinicians immediately after each consultation encouraged reflection of the use and relevance of the CDSS for individual cases. Longer interviews with clinicians following regular use of the CDSS aimed to explore their overall views of the usefulness of the tool. The observation data provided a means of confirming the accuracy of recall at interview. The focus group allowed for discussion and exchange of views amongst clinicians about the day-do-day benefits and difficulties of using the CDSS and its potential implementation in practice. Purposive sampling was used for clinic observations, to gain a maximum variation sample across all clinicians, who worked on different days of the week. Post-consultation field interviews were conducted where feasible in between appointments.

Qualitative data were collected between July and November 2010, and are summarised in Table 1. Trust B runs two separate chest pain clinics. We were unable to integrate the CDSS at the first of these clinics due to lack of internet facilities within the clinic. As a result the CDSS was only used in the second chest pain clinic. The resultant delay meant that the qualitative researchers were no longer able to observe the introduction of the chest pain clinic in Trust B.

**Table 1:** Qualitative data gathered after delivery of the OMA programme

Data Type	Data type	Number	Sites
Observational	Post-intervention consultations:		Trust A
	Cardiologist	12	
	Specialist cardiac nurse	20	
	Physiologist	8	
		Total: 40	
Self-reported	Field interviews immediately		Trust A
	following observation of CDSS	10	
	Cardiologist	9	
	Specialist cardiac nurse	6	
	Physiologist	Total: 25	
	Clinician interviews	5	Trust A (4) and ^a^Trust B (1)
	Cardiologist	2	
	Specialist cardiac nurse	3	
	Clinician focus group	1	Trust A
	Participants:		
	Cardiologist1		
	Specialist cardiac nurse2		
	Physiologist 1		

^a^Interview carried out with lead clinician at Trust B to explore diversity of issues across sites

Detailed field-notes and audio-recordings were made of all observed consultations, including brief field interviews conducted at the end of consultations. All clinician interviews and the focus group were audio-recorded. Interview and consultation transcripts and observational field-notes were imported into qualitative data analysis software (*atlas.ti* and *Nvivo*), coded and analysed thematically [11]. Four of the investigators (KB, ME, RJ, HC) contributed to the identification of emergent themes.

We linked transcripts and field notes for the observed consultations to clinical information from patients’ medical records and to the recommendations of the CDSS. Insights from the case-by-case analysis of clinicians’ use of the CDSS were synthesised with themes emerging from the analysis of interview and focus group data. Staff are numbered consecutively [S1, S2]. Data collected by interview are indicated by [I]; data collected by observation are indicated by [OB]; field interview by [FI] and focus group by [FG]. . Verbatim quotes are marked with quotation marks.

This study has ethics approval (London-City Road and Hampstead REC and Riverside Research Ethics Committee reference number 08/H0709/85), and research governance approval (from NHS sites).

## Results

### Before and after study

**Fig. 3:**
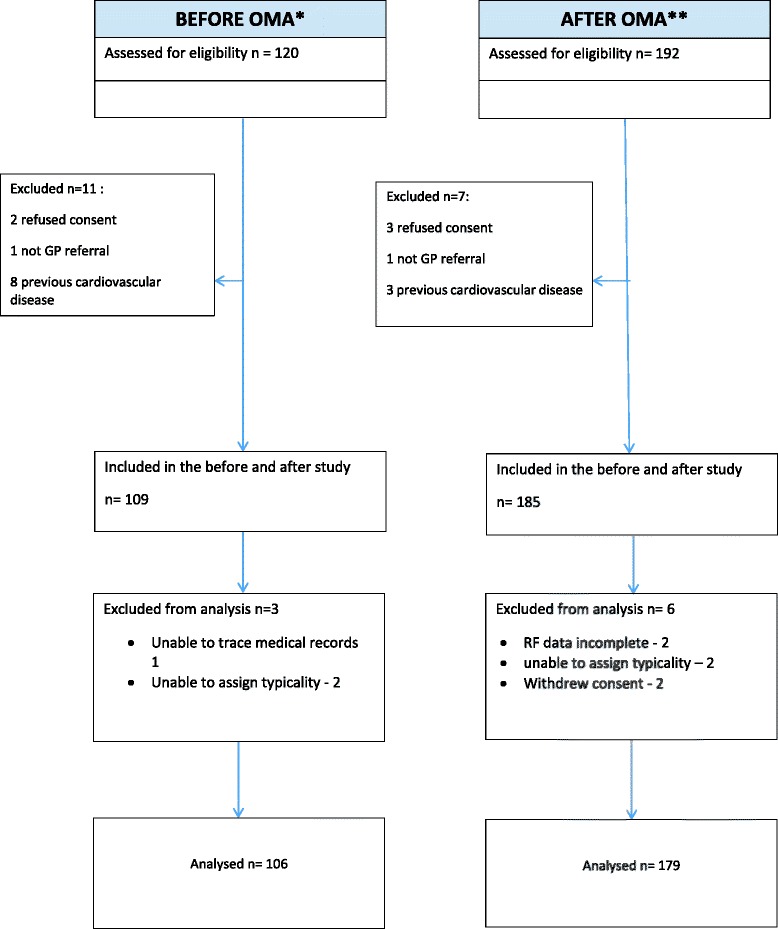
Recruitment to the before and after study

294 patients were recruited to the quantitative study between November 2009 and May 2011; 285 were included in the analysis at six months (Fig. 3): 106 patients seen in a clinic that had not received the OMA programme (*Before OMA*) and 179 patients seen in a clinic that had received the OMA programme (*After OMA*). Characteristics at baseline (chest pain clinic consultation) including cardiovascular risk factors and current medications are shown in Table 2. Clinicians seeing patients in the Before OMA group were cardiologists (3), specialist cardiology nurses (2) and a cardiac physiologist (1), and in the After OMA group were cardiologists (4), specialist cardiology nurses (3) and a cardiac physiologist (1). The CDSS was used for 86 % (154/179 95 CI 81–91 %) of patients in the *After OMA* group. CDSS was used for 81 out of 100 patients (81 %) in Trust A, and 73 out of 79 patients (92 %) in Trust B (difference 11, 95 CI 2–21 %).

**Table 2:** Baseline characteristics at date of chest pain clinic consultation

	BEFORE OMA	AFTER OMA
	N = 106	N = 179
Age Mean (SD)	59.4 (11.0)	59.0 (11.6)
Sex		
Male	52 (49 %)	97 (54 %)
Female	54 (51 %)	82 (46 %)
Risk factors:		
Smoking	43 (41 %)	59 (33 %)
Diabetes	9 (8 %)	20 (11 %)
Hyperlipidaemia	34(32 %)	62 (35 %)
Medications *current at chest pain clinic:*		
Anti-platelet	23 (22 %)	34 (19 %)
Beta-blocker	5 (5 %)	17 (9 %)
Statin	26 (25 %)	37 (21 %)
Typicality of chest pain:		
Typical	20 (19 %)	46 (26 %)
Atypical	17 (16 %)	28 (16 %)
Non-cardiac	69 (65 %)	105 (59 %)

The CDSS recommended investigation in 34 % (95 CI 25–43 %) of patients *Before OMA* and 41 % (95 CI 34–48 %) of patients *After* OMA. Table 3 shows the number of patients having any investigation done within six months, by CDSS recommendation. The proportion of patients for whom the CDSS recommendation was followed was 74 % (95 CI 65–82 %) in the *Before OMA* group and 75 % (95 CI 69–81 %) in the *After OMA* group. The proportion of patients for which the CDSS recommended no investigation that did not have one was the same before and after the OMA intervention. Where the CDSS recommended an investigation, the proportion receiving an investigation was 50 % [CI 34–66 %] in the *Before OMA* group and 59 % [95 CI 48–70 %] in the *After OMA* group. The CDSS recommended an increase in the number of medication classes for 25 patients (24, 95 CI 16–32 %) *Before OMA*. Of these, eight (32, 95 CI 14–50 %) patients received at least one additional medication class, two (8 95 CI −3–19 %) received fewer medication classes and 15 (60, 95 CI 41–79 %) had no change. In the *After OMA* group, the CDSS recommended an increase in the number of medication classes for 34 (19, 95 CI 13–25 %) participants. Of these, 17 (50, 95 CI 33–67 %) were prescribed at least 1 additional medication class, 7 (21, 95 CI 7–35 %) were prescribed at least 1 fewer medication classes and 10 (29, 95 CI 14–44 %) were unchanged.

**Table 3:** Agreement of investigations with CDSS recommendations

	Investigation done		
Before OMA CDSS recommendation (investigation or no investigation) followed in 78 out of 106 cases (74, 95 CI 65–82 %)			
CDSS recommends:-	n	Done	% done (95 % CI)
Investigation	36	18	50 (34–66)
No investigation	70	10	14 (6–22)
After OMA CDSS recommendation (investigation or no investigation) followed in 134 out of 179 cases (75, 95 CI 69–81 %)			
CDSS recommends:-			
Investigation	74	44	59 (48–70)
No investigation	105	15	14 (7–21)

### Qualitative study

**Fig. 4:**
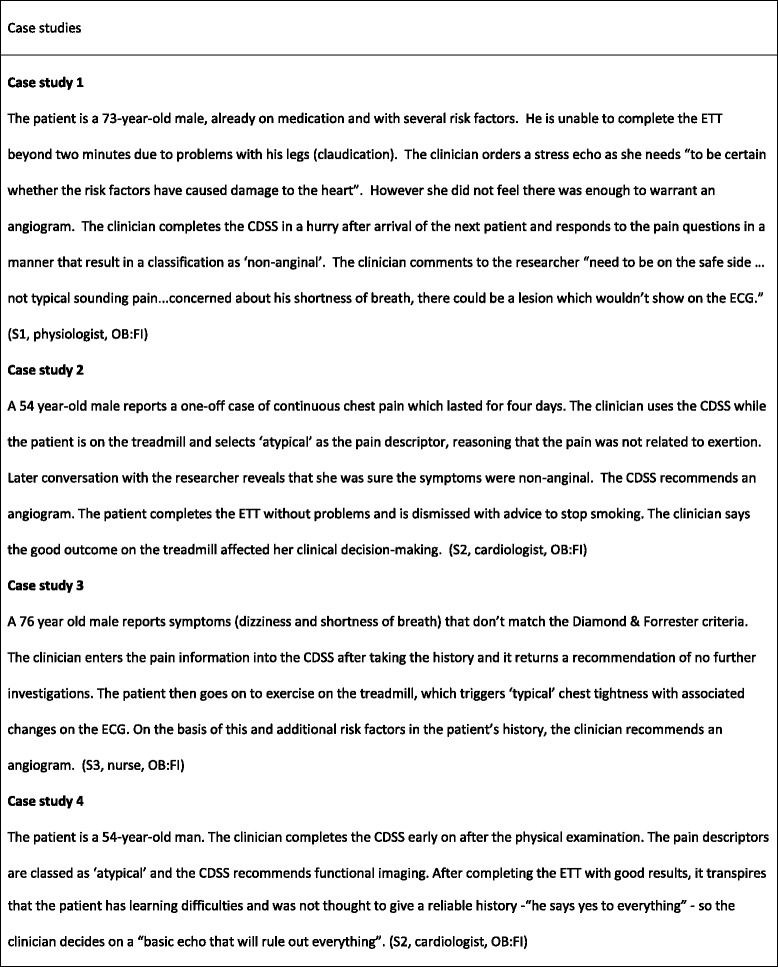
Case studies

**Fig. 5:**
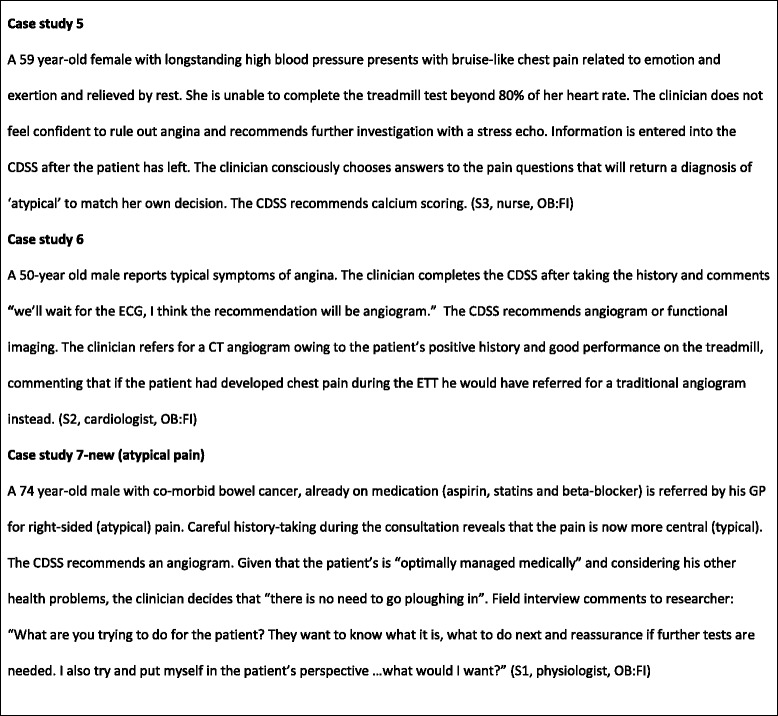
Case studies

Although CDSS use was high, we found that clinicians used the CDSS differently than originally intended. Case studies draw on observational and interview data to illustrate the themes identified and are found in Figs. 4 and 5.

### Problems with the entry of patient information into the CDSS Problems with initial pain categorisation

**Fig. 6:**
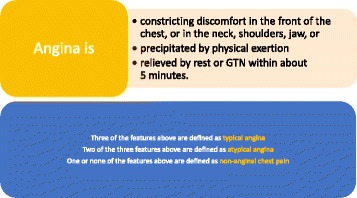
Classification of chest pain. Source: NICE CG95 [9]

**Fig. 7:**
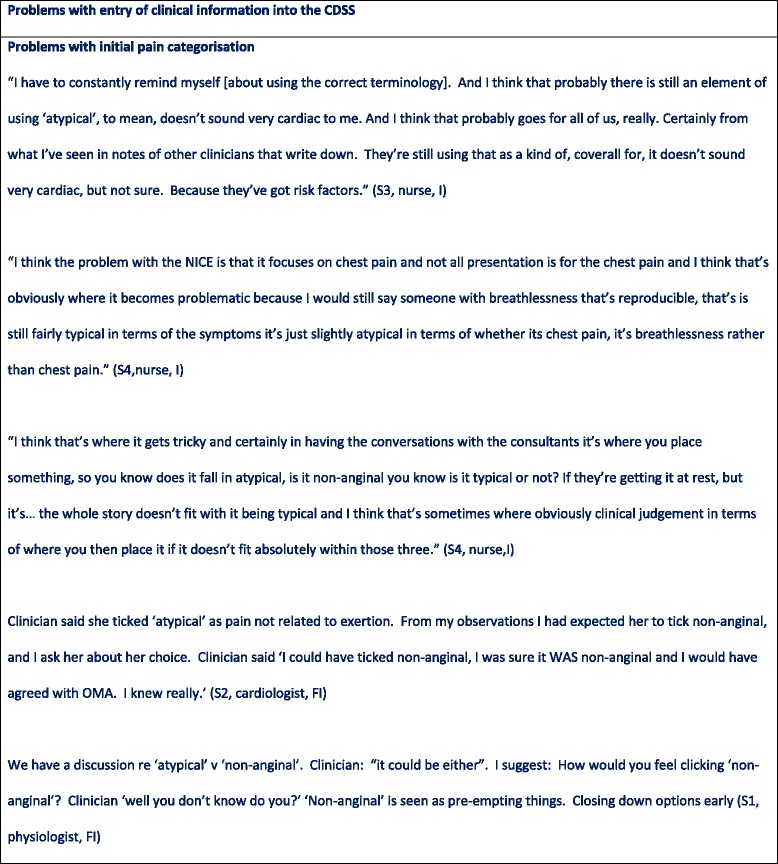
Problems with entry of patient information into the CDSS

**Fig. 8:**
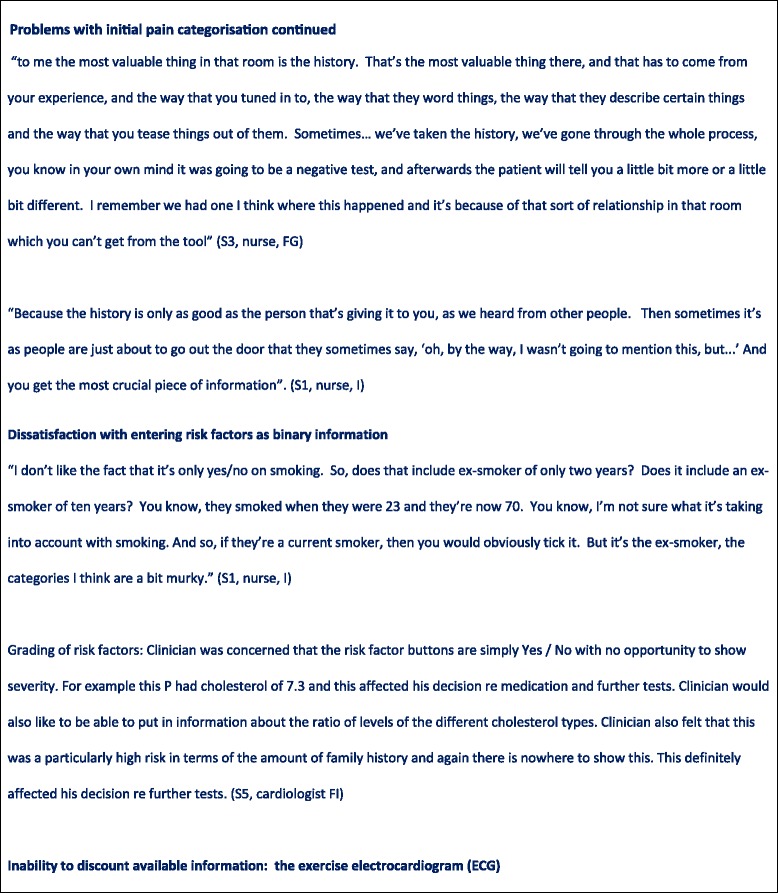
Problems with entry of patient information into the CDSS

**Fig. 9:**
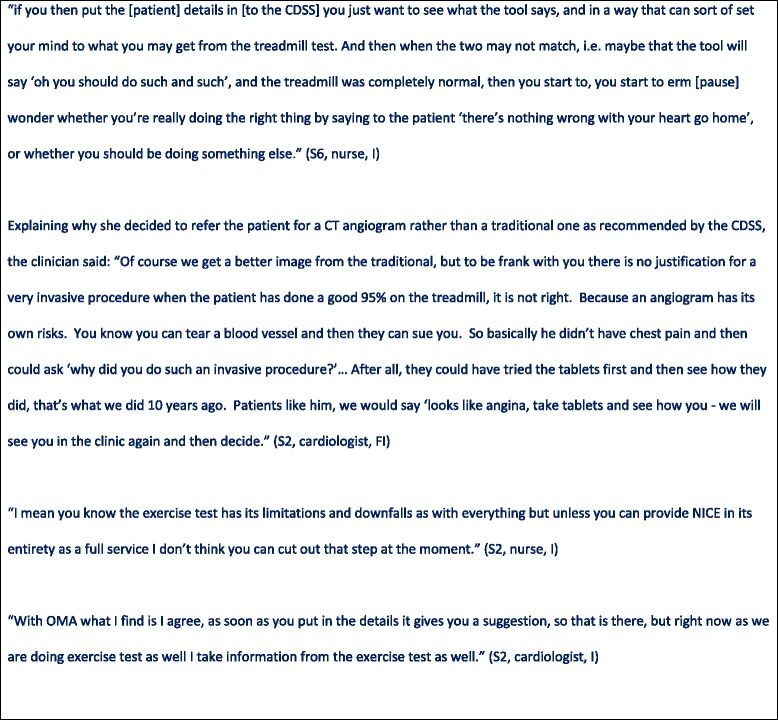
Problems with entry of patient information into the CDSS

In the NICE guideline chest pain is labelled as ‘typical’, ‘atypical’ or ‘non-anginal’ depending on the answers to three questions (Fig. 6) [9]. However, through observation it became clear that clinicians viewed translating patient reported symptoms into these three categories as problematic (see Figs. 4, 5, 7, 8 and 9). Use of the pain labels required a change to their usual practice, and the use of the label ‘atypical’ was frequently problematic. For example, one nurse described how she felt most of the chest pain clinicians used the term ‘atypical’ where pain was not thought to be cardiac but there was uncertainty. Others described a reluctance to use the term ‘non-anginal’ because of its diagnostic certainty, and there were examples of clinicians entering the description ‘atypical’ while saying that they were sure that the pain was non-cardiac. Although one nurse said that in responding to the questions he would try and stick closely to the patient’s report of their symptoms regardless of his own hunches, researchers also observed clinicians consciously answering the pain quality questions in a particular way to match their clinical judgment or to manipulate a particular CDSS response. In some cases, patients’ stories changed during the consultation. Further, clinicians described the skill required in eliciting ‘the whole story’ of chest pain from a patient, of which the three diagnostic questions represented only a part.

### Dissatisfaction with entering risk factors as binary information

Clinicians expressed concern that the CDSS did not allow them to grade information about patients’ cardiovascular risk factors, as they felt entering these factors in binary form (present or absent) omitted important details they had available to them. For example, patients with particularly high cholesterol levels were considered to be at higher risk, yet this level of detail was not captured by the CDSS. Although the clinicians understood that the algorithm underlying the CDSS was based on diagnostic and prognostic research evidence, this did not reflect the way in which they understood and weighted risk factors in practice. For example, clinicians expressed frustration that risk factors that they routinely used in clinical practice, in particular family history, did not affect the CDSS recommendations.

### Inability to discount available information: the exercise electrocardiogram

During the study, the treadmill exercise electrocardiogram (exercise tolerance test, ETT) was still used to assess most patients. However, in line with NICE guidance, the CDSS algorithm did not incorporate the results of the ETT. Clinicians were unwilling to ignore additional information from the ETT and one clinician explained that she would use the CDSS only after she had interpreted the ETT so as to not ‘cloud her judgment’. At the same time, they acknowledged the limitations of the ETT’s diagnostic value. Case studies 3 and 4 provide examples of clinicians’ interpretations of the patient history changing during the course of the consultation. Case studies 2, 5 and 6 are examples where patients’ ETT performance contributed to clinicians’ decision to deviate from CDSS recommendations.

### Structural and practical barriers to using the CDSS as intended

Structural barriers to implementing the CDSS recommendations included inability of clinicians other than doctors to prescribe medication, and the unavailability of specific imaging methods at the hospital site. Authorised prescribers rarely made use of the available facility for direct issuing of medication from the hospital pharmacy. Reasons cited for this were clinicians not being in the habit of prescribing (it was their usual practice to issue recommendations to the patient’s GP), GPs having a better grasp of how medication fitted with patients’ existing drug regimen and additional cost to the hospital trust. Clinicians chose readily accessible investigations over those with limited availability (CT angiography) or longer waiting times (stress echocardiography) at the time of the study; this often led to choosing different investigations to those recommended by the CDSS. Clinicians also had to complete separate electronic records with similar information to that requested by the CDSS, which posed a competing demand and felt like duplication of effort.

### Impact of the CDSS on clinicians’ diagnostic decision–making and patient management

**Fig. 10:**
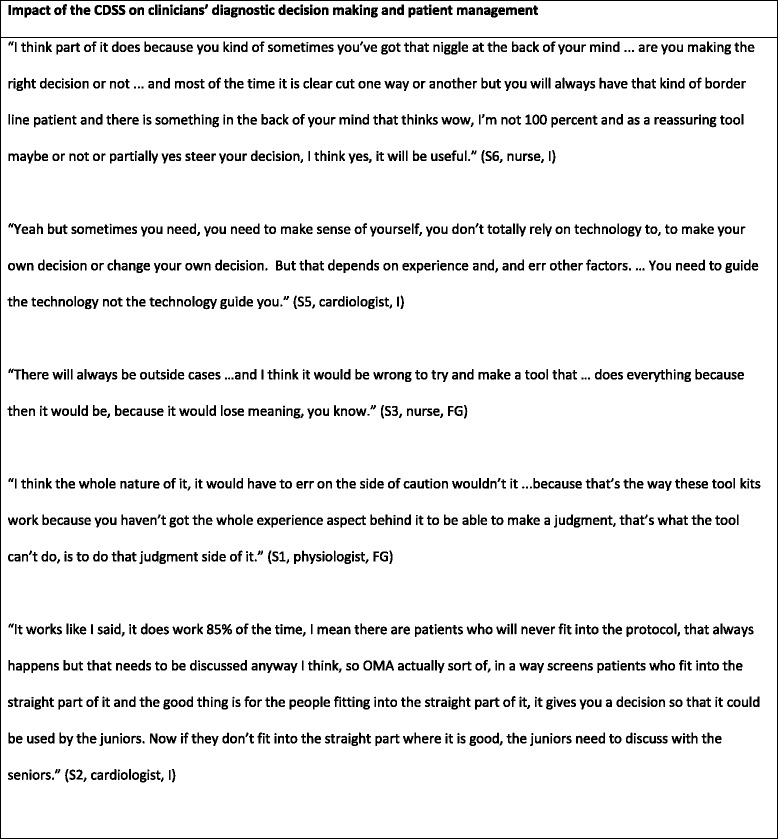
Impact of the CDSS on diagnostic decision-making and patient management

The impact of the CDSS on diagnostic decision making and patient management are illustrated in Figs. 4 and Fig. 10. Clinicians disagreed with CDSS recommendations about investigative procedures in almost half of all observed cases. Clinician disagreement encompassed both more and less investigations than recommended by the CDSS. Researcher observations confirmed that, in all these cases, clinicians stuck to their original decision instead of considering changes in light of the CDSS recommendations. Indeed, there was a broad consensus amongst clinicians articulated in interviews and the focus group that clinical decision-making could not and should not be driven by technology and that clinicians should remain final arbiters of any decisions to be implemented. They described the possible benefits to themselves of using the CDSS mainly in terms of providing reassurance and supporting a systematic approach to conducting consultations. This orientation towards the CDSS as ‘decision feedback’ rather than ‘decision support’ was also reflected in the timing of clinicians’ engagement with the CDSS. In two thirds of the observed cases, clinicians used the CDSS *after* they had communicated their decision to the patient and the patient had left the room.

Clinicians were most likely to agree with the CDSS guidance when pain was categorised as ‘non-anginal’, resulting in a recommendation of ‘no further investigation’. For some typical case presentations of angina symptoms clinicians also expressed agreement with CDSS recommendations in theory, though in practice they ‘downgraded’ the recommended methods of investigation in a few cases where they felt that a more aggressive approach was not in the patient’s best interest or clinically unwarranted (see Case studies 6 and 7 for examples). Clinicians were most likely to disagree with the CDSS when they had categorised the patient’s pain as ‘atypical’. In several of these cases the clinician had expressed certainty that the pain was ‘non-anginal’ but had entered it as atypical, leading to a recommendation for further investigation. Often clinicians were swayed by the presence of marked risk factors to investigate patients in the face of recommendations not to do so. In many cases the results of the exercise test were observed to influence the decision to investigate further. Case studies 1,2,3, and 5 provide examples of such instances of disagreement. Clinicians regarded CDSS recommendations as most reliable and trustworthy - but probably also least needed - when case histories were clearly typical for angina or clearly non-anginal.

## Discussion

Our study contributes new findings to the understanding of how a CDSS is used in the initial management of a common symptom. Despite use of the OMA CDSS in 86 % of consultations for which it was available, there was little evidence of impact on medication prescribing or on investigation choice. Through qualitative data analysis we gained an in-depth understanding of the reasons for this discrepancy. We identified problems in entering patient data into the CDSS. These included difficulties in classification of symptoms and risk factors, and in the incorporation of all available clinical information emerging during the consultation. In the majority of observed cases, the CDSS was used after the patient had left the room. Structural and practical barriers to the use of the CDSS included the availability of investigations and the prescribing competencies of the clinicians. Analysis of observational data revealed how clinicians privileged their clinical expertise over CDSS advice, responding to CDSS recommendations as ‘decision feedback’ rather than ‘decision support’. There was most disagreement with CDSS recommendations for patients whose pain was categorised as atypical, the group for which mapping patient symptoms to the CDSS had proved most challenging. Chest pain clinicians were rarely observed prescribing medication from the clinic. Taken together these data indicate little chance of the OMA CDSS influencing clinical outcomes, and as such we will not proceed to a cluster randomized controlled trial of the OMA CDSS.

Observing consultations in a chest pain clinic, Somerville and colleagues [12] found that doctors were engaged in practical interpretive work, actively restructuring patient narratives to meet an accepted diagnostic classification. Pain narratives were sometimes unstable and changing, and symptoms outside the canon were investigated less frequently. We have observed clinicians engaged in this interpretive work, but also struggling to use a classification that was sometimes at odds with their usual way of working, despite our efforts during CDSS development to understand clincians’ working practices. Our clinicians worked creatively with the constraints of the tool, in line with Berg’s description of how protocols for care can be circumvented, tinkered with and interpreted in many different ways [13]. We observed clinicians developing ‘workarounds’ to enable the system to be used, introducing the potential for unintended consequences of CDSS use [14]. Previous qualitative studies have identified CDSSs having little impact on decisions either through non-use of the CDSS [15], or use after the consultation has been completed [16]. A study of nurses using CDSS identified how they were primarily used to confirm decisions that had already been made [17].

The OMA CDSS delivered a national clinical guideline through patient specific recommendations. The constraining effects of guidelines have been previously articulated, including ignoring data that cannot easily be measured, and failing to allow the tailoring of advice to individual patients [18, 19]. A major limitation of guidelines is their poor fit to complex clinical problems, particularly in the presence of multi-morbidity, being more suited to relatively straightforward clinical decisions, [20]. From our findings, this limitation extends to CDSSs.

A strength of our study lies in its mixed methods approach. Although the quantitative data suggested that the CDSS was used, the qualitative data were essential in developing a more complete understanding of how it was used. A limitation of the quantitative study was its relatively small sample size, precluding statistical analysis. It is possible that some appropriate investigations may have happened after six months, at which point our follow-up date was censored, so this was a conservative assessment of adherence to investigation recommendations. We have measured medication prescription in general practice six months after the chest pain clinic visit, as a proxy for successful commencement or recommendation of long term secondary prevention medication at the time of the clinic. There are many other factors affecting whether medication is continued at six months, such as emergent indications for medication. Although CDSS use in both trusts was high, it appeared to be significantly different between Trust A and B. A limitation of the qualitative study is that were unable to observe Trust B after the introduction of the CDSS.

The OMA CDSS was introduced at a time of transition for chest pain services, the NICE guidance on which it was based having been published four months earlier. At this time, the ETT test played a central role in chest pain clinic routines, and our main qualitative study site did not routinely use computerised tomography or computer tomography coronary angiography for the diagnosis of angina. The NICE guidance advised against the use of the ETT, and recommended increased numbers of non-invasive tests including computerised tomography investigations. We found that the failure of the guidance to incorporate a test that was an entrenched part of chest pain clinic practice [21] added to the difficulties of the clinicians in using the CDSS. It is possible that the CDSS would have been more able to impact upon clinic processes when chest pain clinics were more able to follow the guidance. We have reported how often clinicians’ choice to investigate agreed with the CDSS recommendations (concordance). Although it might be assumed that concordance with NICE guidance reflects appropriate management, we acknowledge the limitations of concordance in the assessment of CDSS. Concordance may be affected by many and diverse things, for example the structural and practical barriers identified in this study, or the perceived appropriateness of the underpinning guidance for a specific clinical scenario.

## Conclusions

The OMA CDSS, and its underpinning NICE guidance, required contextualised and nuanced clinical information to be categorised in a way that did not fit with clinicians’ ways of working. This, in combination with a belief on the part of participating clinicians that clinical decision making should not be driven by technology, meant that the CDSS did not substantially inform individual patient management. Qualitative methods, proved essential in deepening our understanding of the factors affecting CDSS use. Use of the CDSS may have been improved by the provision of guidance for both practitioners and patients [5] and a more intensive training and monitoring programme [22]. CDSSs for the assessment and management of new onset chest pain may be more successful if they reflect more closely the ways in which clinicians assess chest pain. Our study has articulated barriers to CDSS use that need to be addressed in the design of future interventions.

## Abbreviations

CDSS: clinical decision support system
OMA: Optimising the management of angina
NICE: National Institute for Health and Care Excellence

## References

[1] Bright TJ, Wong A, Dhurjati R, Bristow E, Bastian L, Coeytaux RR (2012). Effect of clinical decision-support systems: a systematic review. Ann Intern Med.

[2] Kawamoto K, Houlihan CA, Balas EA, Lobach DF (2005). Improving clinical practice using clinical decision support systems: a systematic review of trials to identify features critical to success. BMJ.

[3] Hunt DL, Haynes RB, Hanna SE, Smith K (1998). Effects of computer-based clinical decision support systems on physician performance and patient outcomes: a systematic review. Jama.

[4] Lobach DF (2013). The road to effective clinical decision support: are we there yet?. BMJ.

[5] Roshanov PS, Fernandes N, Wilczynski JM, Hemens BJ, You JJ, Handler SM (2013). Features of effective computerised clinical decision support systems: meta-regression of 162 randomised trials. BMJ.

[6] Sekhri N, Feder GS, Junghans C, Hemingway H, Timmis AD (2007). How effective are rapid access chest pain clinics? Prognosis of incident angina and non-cardiac chest pain in 8762 consecutive patients. Heart (British Cardiac Society).

[7] Sekhri N (2009). Rapid Acchess Chest Pain Clinics: Characteristics and outcomes of patients from six centres.

[8] Michie S, Johnston M, Abraham C, Lawton R, Parker D, Walker A (2005). Making psychological theory useful for implementing evidence based practice: a consensus approach. Qual Saf Health Care.

[9] Conditions NCGCfAaC (2010). Chest Pain of Recent Onset: Assessment and Diagnosis of Recent Onset Chest Pain or Discomfort of Suspected Cardiac Origin.

[10] Pryor DB, Shaw L, McCants CB, Lee KL, Mark DB, Harrell FE (1993). Value of the history and physical in identifying patients at increased risk for coronary artery disease. Ann Intern Med.

[11] Braun V, Clarke V (2006). Using thematic analysis in psychology. Qual. Res. Psychol..

[12] Somerville C, Featherstone K, Hemingway H, Timmis A, Feder GS (2008). Performing stable angina pectoris: an ethnographic study. Soc Sci Med.

[13] Berg M (1997). Problems and promises of the protoco. Soc Sci Med.

[14] Ash JS, Berg M, Coiera E (2004). Some unintended consequences of information technology in health care: the nature of patient care information system-related errors. Journal of the American Medical Informatics Association : JAMIA.

[15] Eccles M, McColl E, Steen N, Rousseau N, Grimshaw J, Parkin D (2002). Effect of computerised evidence based guidelines on management of asthma and angina in adults in primary care: cluster randomised controlled trial. BMJ.

[16] Lomotan EA, Hoeksema LJ, Edmonds DE, Ramirez-Garnica G, Shiffman RN, Horwitz LI (2012). Evaluating the use of a computerized clinical decision support system for asthma by pediatric pulmonologists. Int J Med Inform.

[17] Dowding D, Mitchell N, Randell R, Foster R, Lattimer V, Thompson C (2009). Nurses' use of computerised clinical decision support systems: a case site analysis. J Clin Nurs.

[18] Rashidian A, Eccles MP, Russell I (2008). Falling on stony ground? A qualitative study of implementation of clinical guidelines' prescribing recommendations in primary care. Health Policy.

[19] McCormack JL, Ash JS (2012). Clinician perspectives on the quality of patient data used for clinical decision support: a qualitative study. AMIA Annual Symposium proceedings/AMIA Symposium AMIA Symposium.

[20] Guthrie B, Payne K, Alderson P, McMurdo ME, Mercer SW (2012). Adapting clinical guidelines to take account of multimorbidity. BMJ.

[21] Cramer H, Evans M, Featherstone K, Johnson R, Zaman MJ, Timmis AD (2012). Treading carefully: a qualitative ethnographic study of the clinical, social and educational uses of exercise ECG in evaluating stable chest pain. BMJ Open.

[22] Unverzagt S, Oemler M, Braun K, Klement A (2014). Strategies for guideline implementation in primary care focusing on patients with cardiovascular disease: a systematic review. Fam Pract.

